# A preliminary attempt to investigate mirror self-recognition in *Octopus vulgaris*

**DOI:** 10.3389/fphys.2022.951808

**Published:** 2022-08-30

**Authors:** Piero Amodio, Graziano Fiorito

**Affiliations:** Department of Biology and Evolution of Marine Organisms, Stazione Zoologica Anton Dohrn, Napoli, Italy

**Keywords:** mirror, self-recognition, octopus, cephalopod, mark test

## Abstract

Mirror self-recognition (MSR) is a potential indicator of self-awareness. This capability has been widely investigated among vertebrates, yet it remains largely unstudied in invertebrates. Here we report preliminary data about behavioural responses exhibited by common octopuses (*Octopus vulgaris*) toward reflected images of themselves and explore a procedure for marking octopus’ skin in order to conduct the Mark test. Octopuses (*n* = 8) received four familiarization trials with a mirror and four familiarization trials with a control stimulus: a non-reflective panel (*Panel* group, *n* = 4) or the sight of a conspecific housed in an adjacent tank (*Social* group, *n* = 4). Subsequently, octopuses were marked with non-toxic nail polish in the area where the Frontal White Spots are usually expressed, and they received one test trial with the mirror and one control trial with no mirror. We found that octopuses in the *Panel* group tended to exhibit a stronger exploratory response toward the mirror than the non-reflective panel, but performed agonistic responses only in the presence of the mirror. In contrast, octopuses in the *Social* group exhibited comparable exploratory and agonistic behaviours toward the mirror and the sight of the conspecific. In the Mark test, octopuses frequently explored the mark *via* their arms. However, mark-directed behaviours were also observed in the absence of the mirror and in sham-marked individuals, thus suggesting that proprioceptive stimuli drove these responses. Despite the limitations associated with our marking procedure, the baseline data collected in this pilot study may facilitate the further testing of MSR in the octopus and other cephalopods.

## Introduction

Mirrors are popular tools in the field of animal behaviour and cognition. Classically used to investigate agonistic behaviour (e.g., Lissmann, 1932; Tinbergen, 1951), reflective surfaces have acquired a prominent role in the study of self-awareness since Gallup (1970) devised the so-called “Mark test” to probe mirror self-recognition (MSR) in chimpanzees. In short, individuals are confronted with their reflected image after a dye has been applied on a part of the body that can only been seen by the animal itself through the mirror. It is assumed that passing the Mark test—i.e., exhibiting mark-directed behaviours only in front of the mirror and towards visible marks, but not sham marks—is evidence that the individual can recognize its reflection. Consequently, this is considered an indicator of self-awareness (Gallup, 1970). Note, however, that alternative accounts have challenged the relationship between successful performances in the Mark test, MSR, and self-awareness (e.g., Heyes, 1994; Mitchell, 1997) or evoked a more gradualist approach to studying self-awareness and interpreting the performance of non-human animals in the Mark test (de Waal, 2019; Wittek et al., 2021).

Studies testing MSR involve a preliminary phase of familiarization in which unmarked individuals are allowed to freely interact with a mirror. Those that pass the test typically exhibit different kinds of response in a progressive fashion during the familiarization phase. In particular, 1) social responses such as agonistic displays, and 2) physical exploration of the mirror and the area behind precede 3) contingency checking, i.e., unusual repetitive body movements; finally, 4) self-exploratory behaviours (e.g., inspection of body parts that can only be seen in a mirror) are exhibited (Plotnik et al., 2006; Brecht et al., 2020). Therefore, performance in the familiarization phase may provide important indications about whether mirror reflections are interpreted as the image of a conspecific or the individual itself (Anderson and Gallup, 2015).

More than 50 years after Gallup’s (1970) study, MSR has been widely investigated in vertebrates, from primates to fish. In addition to the great apes (Gallup, 1970; Suarez and Gallup, 1981; Anderson and Gallup, 2015), only a few species have been claimed to have passed the Mark test, most notably dolphins (Reiss and Marino, 2001), elephants (Plotnik et al., 2006), magpies (Prior et al., 2008), and cleaner wrasses (Kohda et al., 2019, 2022). However, evidence of MSR in non-primates remains controversial, typically due to the difficulties in interpreting the performance in the Mark test by animals whose morphologies constrain clear attempts to remove the mark or methodological issues with the marking procedure and design (Gallup and Anderson, 2018, 2020). For instance, Prior et al. (2008) used stickers to mark magpies, a procedure that might have provided tactile stimuli to the birds, therefore confounding the results (Soler et al., 2014). A more cautious interpretation of MSR capabilities in magpies is further justifed by the negative results obtained in the same species (Soler et al., 2020) and in other members of the corvid family (e.g., carrion crows, Brecht et al., 2020; Vanhooland et al., 2020; for reviews in corvids see Baciadonna et al., 2021; Brecht and Nieder, 2020). In contrast to the foregoing studies in vertebrates, MSR remains largely unexplored in invertebrates (but see Riojas-Schnier and Toth, 2022 for a recent experiment in wasps). Coleoid cephalopods (cuttlefish, squid and octopus) represent a valuable candidate invertebrate group for addressing this gap in a systematic fashion. First, these molluscs exhibit complex nervous systems (Young, 1971, 1991; Shigeno et al., 2018) and flexible behavioural repertoires (reviewed in: Amodio et al., 2019b, 2019a; Hanlon and Messenger, 2018), two features that may indicate a certain degree of cognitive sophistication (for a critical discussion see: Amodio, 2019; Schnell et al., 2021; Ponte et al., 2022). Second, coleoids evolved not only acute vision (reviewed in: Budelmann, 1995), but also visual systems that are specialized for detection of transient skin markings such as stripes and spots, to mention but a few. Adaptations in coleoid visual systems clearly evolved, at least in part, for the perception of dynamic body patterning exhibited through the skin which afforded the rich repertoires of bodily appearance used as signals to communicate with conspecifics and heterospecifics (for review see: Borrelli et al., 2006; Hanlon and Messenger, 2018). Third, coleoids are equipped with sets of flexible appendages that can be employed to physically explore their own bodies as well as the external environment. Octopuses regularly groom their heads and mantles through their arms (Mather, 1998). Importantly, experimental evidence also indicates that octopuses can guide the movement of one arm toward a specific location using visual information (Gutnick et al., 2011). Therefore, the anatomical, behavioural, and sensory adaptations of coleoids seem particularly well suited to the detection of skin marks and the expression of mark-directed responses, both of which are crucial requirements for passing the Mark test. Nevertheless, the investigation of MSR in coleoids poses unique—albeit not intractable—challenges. The lack of fur, feathers, or scales on the bodies of these animals, together with their colour changing ability, make the marking procedure more complicated than in the case of other species.

Despite the promise of coleoids as good candidates for the study of MSR, little is known about how these molluscs perceive and respond to reflected images of themselves; nevertheless, some possible indications of MSR have been collected in the context of visual communication. Examining the role of polarization vision in intraspecific interactions, Shashar et al. (1996) found that short exposures (e.g., up to 30 s) to mirrors induce common cuttlefish (*Sepia officinalis*) to retreat from images of themselves. In the same species, Palmer et al. (2006) observed a previously undescribed body pattern (termed “*Splotch*”) displayed only by female cuttlefish in the presence of mirrors and same-sex conspecifics, thus suggesting that reflected self images may have been perceived as another individual of the same sex. In addition, a few preliminary investigations by Ikeda and colleagues have employed mirrors to explore MSR in coleoids (for a review see Ikeda, 2009). In one experiment, Ikeda and Matsumoto (2007) presented a group of squid (*Sepioteuthis lessoniana*) with a mirror and a wood board. They found that the former induced some individuals of the school to approach and physically explore the stimulus, whereas the latter did not alter the schooling behaviour relative to when no stimulus was present. In a subsequent study, Ikeda and Matsumura (2008) extended testing of mirror-induced response to seven additional species, including a cuttlefish (*Sepia pharaonis*) and several octopus species (e.g., *Octopus laqueus*, *Hapalochlaena lunulata*, *Abdopus aculeatus*). The authors reported that squid and cuttlefish showed, respectively, strong and moderate interest towards reflected images of self (e.g., physical exploration, agonistic response), while octopuses did not react to the stimulus (Ikeda, 2009). Finally, a preliminary Mark test experiment in *S. lessoniana* by Ikeda (2007) indicated that individuals with visible marks showed a stronger tendency to observe and physically interact with the mirror, relative to sham-marked individuals (Ikeda, 2009). Unfortunately, the latter two studies have only been published as conference abstracts, so no detail is provided regarding methods and results.

Here, we report a pilot study exploring MSR in the common octopus (*Octopus vulgaris*). In particular, we aimed to: 1) acquire preliminary data about the behavioural response exhibited by octopuses toward a mirror as well as that in response to two control stimuli, namely, a non-reflective panel and a conspecific; and 2) test a procedure for marking octopus’ skin in order to conduct the Mark test.

## Methods

### Subjects and housing

Eight common octopuses (*Octopus vulgaris*) of both sexes (5 M, 3 F) were included in the study (body weight range: 154–406 g). The animals were caught by artisanal fishermen in the Gulf of Naples (Mediterranean Sea, Italy) and transferred to the Stazione Zoologica Anton Dohrn, within a few hours of capture. Octopuses were housed in standardized tanks (60 × 100 × 50 cm) comprised of dark grey PVC, with the front side consisting of a glass panel (45 × 35 cm) to permit video recording of experiments. Following Fiorito and co-workers (e.g., Fiorito and Scotto, 1992; Amodio and Fiorito, 2013; Borrelli et al., 2020), adjacent tanks were separated by a clear lateral wall made of glass which could allow visual—but not physical—interaction between two individually-housed octopuses. This clear lateral wall could be obscured via a removable dark grey PVC panel to prevent visual interaction between pairs of octopuses outside a specific testing condition (see Procedures). A thin layer of sand covered the bottom of the tanks and two bricks, arranged as a den, were placed in the back corner of each tank, opposite the adjacent tanks (Borrelli et al., 2020). Circulating seawater (a semi-open system) was pumped directly from the Gulf of Napoli through the Stazione Zoologica life support systems. Lamps (Neodymlite dichroic halogen MR16, Oy Airam AB, Finland) were positioned at 1.40 m above the tanks and programmed to reproduce the appropriate seasonal dark-light daily cycle at the local latitude (for details see: Borrelli et al., 2020). Animals were fed every other day with a live crab (*Carcinus mediterraneus*). The present study was conducted in November 2012.

### Acclimatization

Octopuses were acclimatized to the laboratory prior to the start of the experiment. In accordance with previous studies (e.g., Amodio et al., 2014; Borrelli et al., 2020), we used octopus’ predatory response as a proxy to evaluate the level of acclimatization. Following the day of arrival in the laboratory, animals were presented each morning with a live crab attached to a cotton thread. The crab generally started to move spontaneously after reaching the bottom of the tank, but if necessary (i.e., in case the crab remained still or exhibited freezing behaviour), the experimenter induced movements by the crab through gentle pulling of the thread. The prey was promptly pulled out of the tank just before the octopus could seize it. We measured the *Latency to attack*—e.g., “the time elapsed from the appearance of the crab at the water surface to just before the octopus’ final pounce on the prey” (Borrelli, 2007, p. 82). Octopuses recovered their predatory response and readily attacked the crab (*Latency of attack* < 10 s) within approximately 1 week. As the predatory response is also considered a measure of the overall motivation and wellbeing of the octopus (Fiorito et al., 2015), we continued to monitor the *Latency of attack* in acclimatized animals daily throughout the study.

### Experimental procedure

The experiment encompassed a familiarization phase followed by the Mark test. During the familiarization phase, octopuses received four trials in which they were exposed to a glass mirror (80 × 50 cm) and four trials in which they were exposed to a control stimulus. The animals were randomly assigned to two groups that differed in the type of control stimulus. The *Panel* group (*n* = 4) was presented with a non-reflective dark grey PVC panel (80 cm × 50 cm), whereas the *Social* group (*n* = 4) was presented with the sight of a conspecific of the same sex and comparable size (e.g., body weight difference <35 g) that was housed in the adjacent tank. This allowed only visual—but not physical—interactions between animals. Octopuses in the *Social* group were exposed to the same individual throughout the familiarization phase.

On each trial, the experimenter waited for the octopus to be inside or in close proximity to the den before presenting the stimulus. The mirror and non-reflective panel were introduced at a distance of approximately 30 cm from the entrance of the den and placed such that the stimulus leaned on the opposite long side of the tank, creating an angle of approximately 20°. This ensured that the octopus could see the stimulus from the den and could explore the area behind the stimulus. To allow visual interaction between animals in the *Social* group, the experimenter lifted the opaque partition obscuring the glass wall between the two tanks, thereby allowing the octopus to see the conspecific housed in the adjacent tank. After 20 min following the presentation of the stimulus, the experimenter introduced a crab attached to a thread into the tank and tested the octopus’ response toward the prey following the same procedure described earlier. In light of limited interaction with mirrors reported for other octopods (Ikeda and Matsumura, 2008; Ikeda, 2009), this aspect of our experimental design was intended to induce the octopus to leave the den and thus increase the chances that the animals would interact with the stimulus. Finally, 30 min after the start of trial, the stimulus was removed and the initial conditions restored.

The familiarization phase was conducted during two consecutive days. On each day, octopuses participated in two sessions (two trials per session), one in the morning and one in the afternoon, approximately 1.5 h after the first session. The order of presentation of stimuli was kept constant such that within each session, octopuses performed the trial with the control stimulus (i.e., non-reflective panel: *Panel* group; conspecific: *Social* group) before performing the trial with the mirror. The inter-trial interval within each session was set to 30 min.

The Mark test was conducted on the third day of the study. In the morning, the octopus was transferred to a bucket filled with a mild anaesthetic solution: seawater with 1.5% MgCl_2_ (Grimaldi et al., 2007). After the animal was sedated, it was marked in correspondence to the Frontal White Spots (Packard and Sanders, 1971; review in Borrelli et al., 2006): two oval spots that are transiently expressed approximately 1 cm below the eyes. This area was selected because it cannot be seen directly without a mirror and because the Frontal White Spots are a salient component of different body patterns (Packard and Sanders, 1971) that might also play a role in intraspecific communication. As a mark, we first applied a drop of Histoacryl® (Aesculap AG)—a soft tissue adhesive—and then, on top of this, a drop of non-toxic nail polish (Frais Monde ®). Five animals were marked with red polish, whereas the remaining three animals received a sham mark (i.e., transparent polish). After the marking procedure was completed, the octopus was moved back to the tank and given one hour to recover. Finally, the animal received two test trials. In the first trial, octopuses in the *Panel* group were presented with the non-reflective panel, whereas octopuses in the *Social* group were presented with no stimulus. In the second trial, all octopuses were presented with the mirror. Test trials were conducted following the same procedure described earlier for the familiarization phase. Inter-trial interval was again set to 30 min. All trials were video-recorded and subsequently analysed.

### Data analysis

**FIGURE 1:**
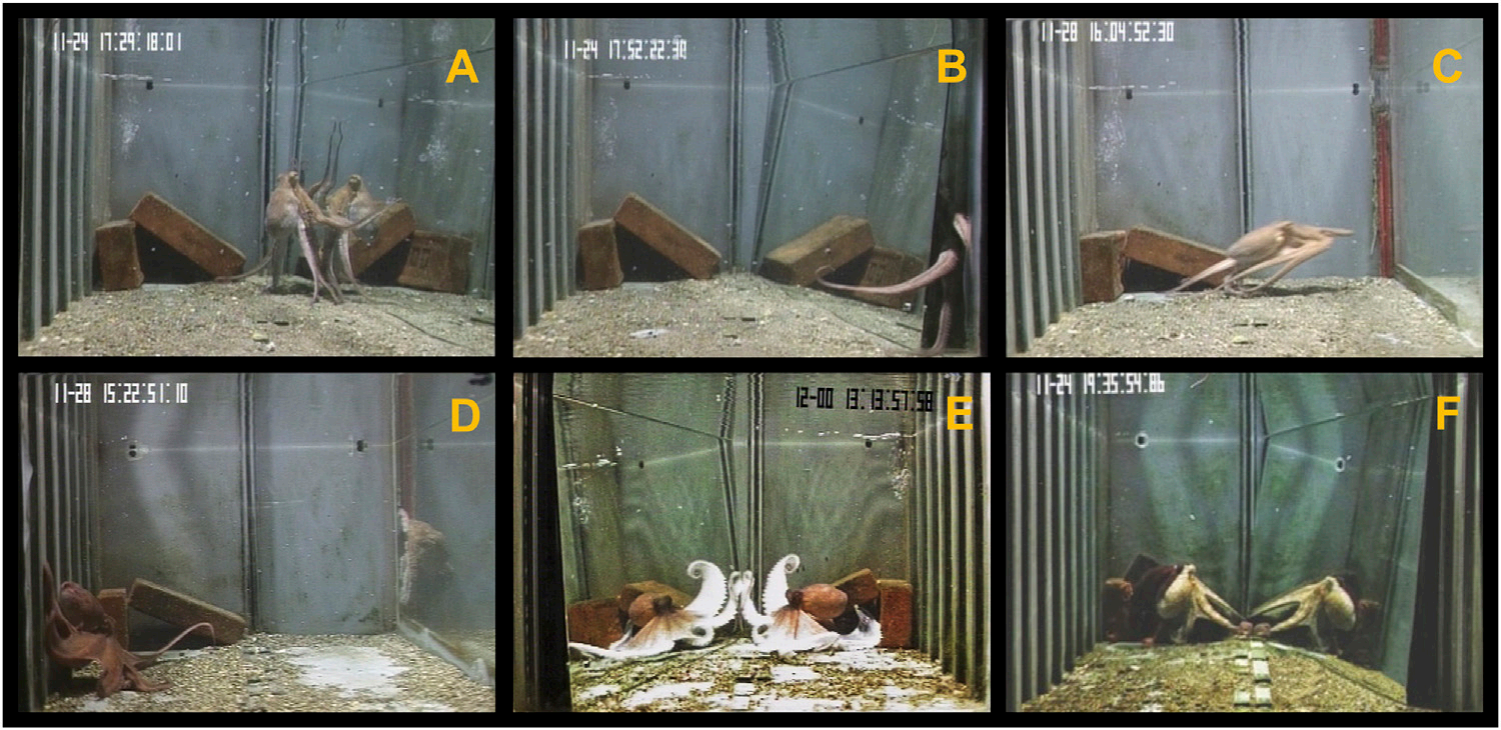
Octopus’ response toward the mirror **(A,B,E,F)** and the conspecific **(C,D)**. Behaviors recognized: **(A)** *Physical contact*; **(B)** *Behind stimulus*; **(C)** *Attack*; **(D)** *Active avoidance*; **(E)** *Bishop*; **(F)** *Unilateral*.

In the familiarization phase, octopus’ response towards the stimuli was characterized in terms of exploratory, agonistic, and self-directed behaviours. We scored eight variables belonging to these three categories (see Table 1; Figure 1). Additionally, we focused on two body patterns that are exhibited in a variety of contexts, namely *Unilateral* and *Passing cloud* (Table 1). It was not possible for the coder to be blind to the conditions during the analysis of video recordings because the stimuli presented to the octopus (i.e., mirror, non-reflective panel, conspecific) were visible in the video recordings.

**TABLE 1:** Definitions of the behavioural variables that were scored in the study.

Category	Behavioural variable	Definition	References
Exploration	*Latency to Touch*	Time elapsed from the moment when the stimulus contacted the surface of the water (or when the partition separating the adjacent tanks was being raised) to the moment of the first contact between the octopus and the stimulus (or the glass side separating the adjacent tanks)	
	*Physical Contact*	Duration of time spent in physical contact (via one or multiple arms) with the mirror, non-reflective panel, or glass side separating the two adjacent tanks. See also Figure 1A	
	*Behind Stimulus*	The octopus explores the area of tank hidden behind the mirror or non-reflective panel. See also Figure 1B	
Agonistic	*Attack*	The “octopus launches itself directly towards the … stimulus, swimming by the propulsion of water from its funnel … and without touching the bottom” (p. 39). See also Figure 1C	Packard, (1963)
	*Active Avoidance*	The octopus maximises the distance between its body and the stimulus, while typically moving away from it and displaying a uniform dark brown coloration. See also Figure 1D	
	*Bishop*	The octopus exhibits curved arms with interbrachial web maximally spread and mantle rounded, often pointed upwards. The animal is dark brown with typically paling arms. See also Figure 1E	Borrelli et al., (2006)
Self-directed	*Grooming*	The octopus “uses one to two arms and bends its arms tubes vertically and laterally so the distal halves extend over its head and mantle and even inside the mantle cavity, generally moving laterally and unevenly in a wormlike motion” (p. 313)	Mather, (1998)
	*Cleaning Manoeuvre*	The movement consists of a “rapid twirling of the arms while they are held in close to the sides of the body starting at the base and continuing with increasing speed to the tips. The keratinuos linings to the suckers are shed in the process and subsequently blown away from the animal by jets from the funnel” (p. 785)	Packard and Sanders, (1971)
Other	*Unilateral*	The animal “may ... be in different phase on one side of the body from that on the other: one side dark, the other light” (p. 93). See also Figure 1F	Packard and Sanders, (1969)
	*Passing Cloud*	A localized “dark flush (lasting less than a second) that passes outwards from the head over [the] dorsal region of arms and web” (p. 785). See also Supplementary Video S1	Packard and Sanders, (1971)

For each variable, we calculated individual mean duration (for *Latency to touch* and *Physical contact*; see Table 1) or frequencies (for all other variables; Table 1) by averaging the response towards each stimulus in the four familiarization trials. Subsequently, we used paired Wilcoxon signed-rank tests to compare the two conditions within each group. Alpha was set to 0.05. In the test, we scored the number of touches to marks performed in each trial. These data were reported descriptively. In addition, as in the case of the familiarization trials, we scored the behavioural variables described in Table 1. These data were not analysed statistically, but rather were collected and described as potentially informative for future studies. All statistical analyses were performed in R (v. 4.0.2) using the RStudio (v. 1.4.1103) wrapper (RStudio Team, 2018).

## Results

### Familiarization: *Panel* group

**FIGURE 2:**
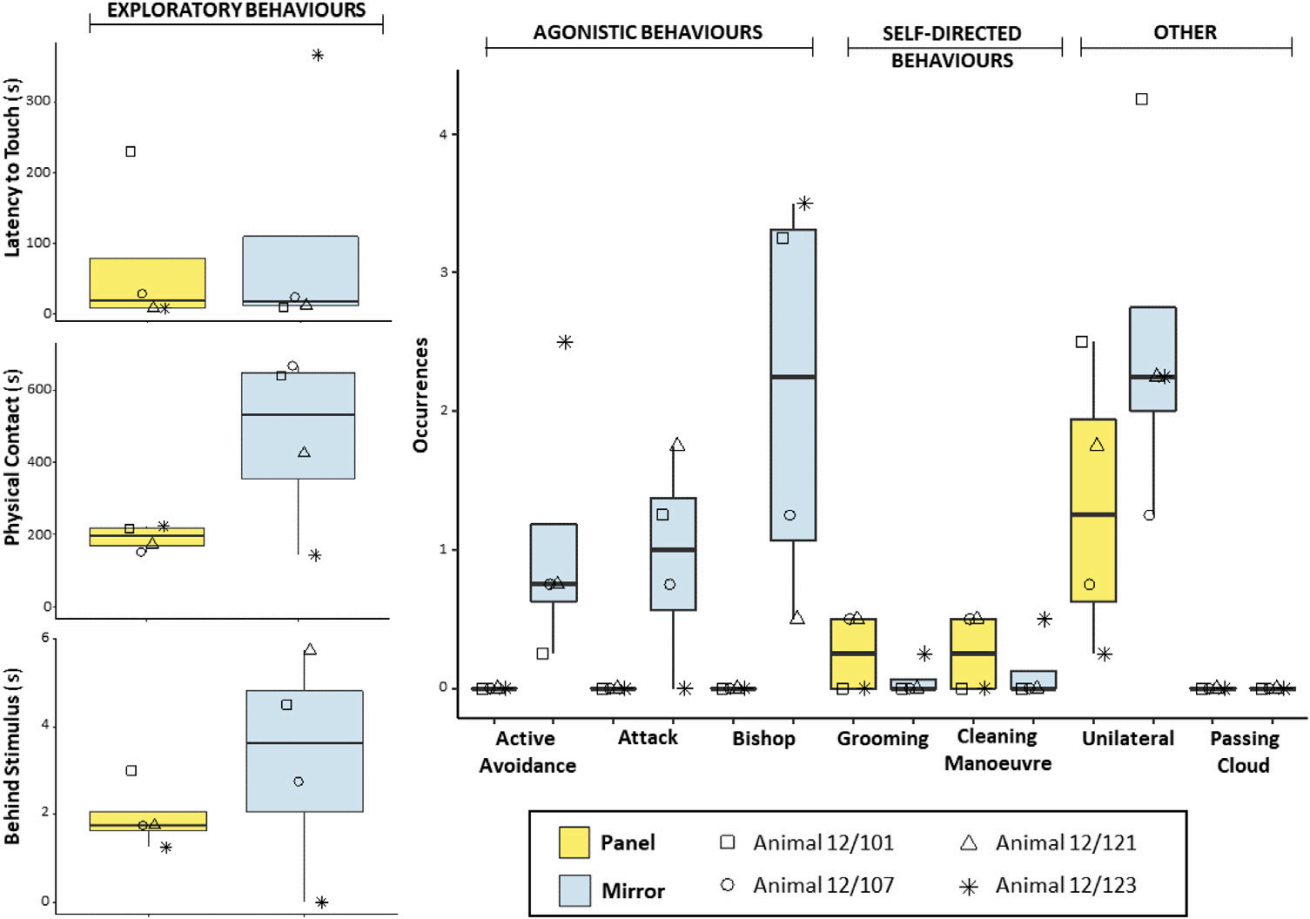
Box and whisker plot of the responses of *O. vulgaris* (*Panel* group) to the mirror (light blue) and the non-reflective panel (yellow) during the familiarization phase.

In the *Panel* group, no significant difference was detected in the comparisons of exploratory behaviours between conditions (Wilcoxon signed-ranked test: *Latency to touch*, *n* = 4, W = 0, *p* = 1; *Physical contact*, *n* = 4, W = 8, *p* = 0.250; *Behind stimulus*, *n* = 4, W = 6, *p* = 0.375). However, octopuses exhibited longer *Physical contact* and more frequent *Behind stimulus* behaviours toward the mirror than toward the non-reflective panel (Figure 2). This trend was also evident at the individual level for all octopuses except one (i.e., Animal 12/123). Agonistic behaviours were exhibited exclusively in the presence of the mirror (Figure 2), therefore no statistical test was conducted to compare the two conditions. Self-directed behaviours were observed at relatively low frequency in the presence of both stimuli (Figure 2). No statistical difference was found in the comparisons of self-directed behaviours between conditions (Wilcoxon signed-ranked test: *Grooming*, *n* = 4, W = −4, *p* = 0.414; *Cleaning Manoeuvre*, *n* = 4, W = −2, *p* = 0.772). Octopuses also exhibited the *Unilateral* body pattern. Despite the fact that discernible behaviours were observed more frequently in trials with the mirror than in trials with the non-reflective panel (Figure 2), comparison of the two conditions yielded no significant difference (Wilcoxon signed-ranked test: *n* = 4, W = 10, *p* = 0.097). Finally, the *Passing cloud* display was never observed in either condition.

### Familiarization: *Social* group

The video footage of one trial was lost (octopus 12/111, trial 2, Social condition) before it could be analysed. For this individual, mean values for the Social condition were therefore calculated by averaging the performance across the three available trials.

**FIGURE 3:**
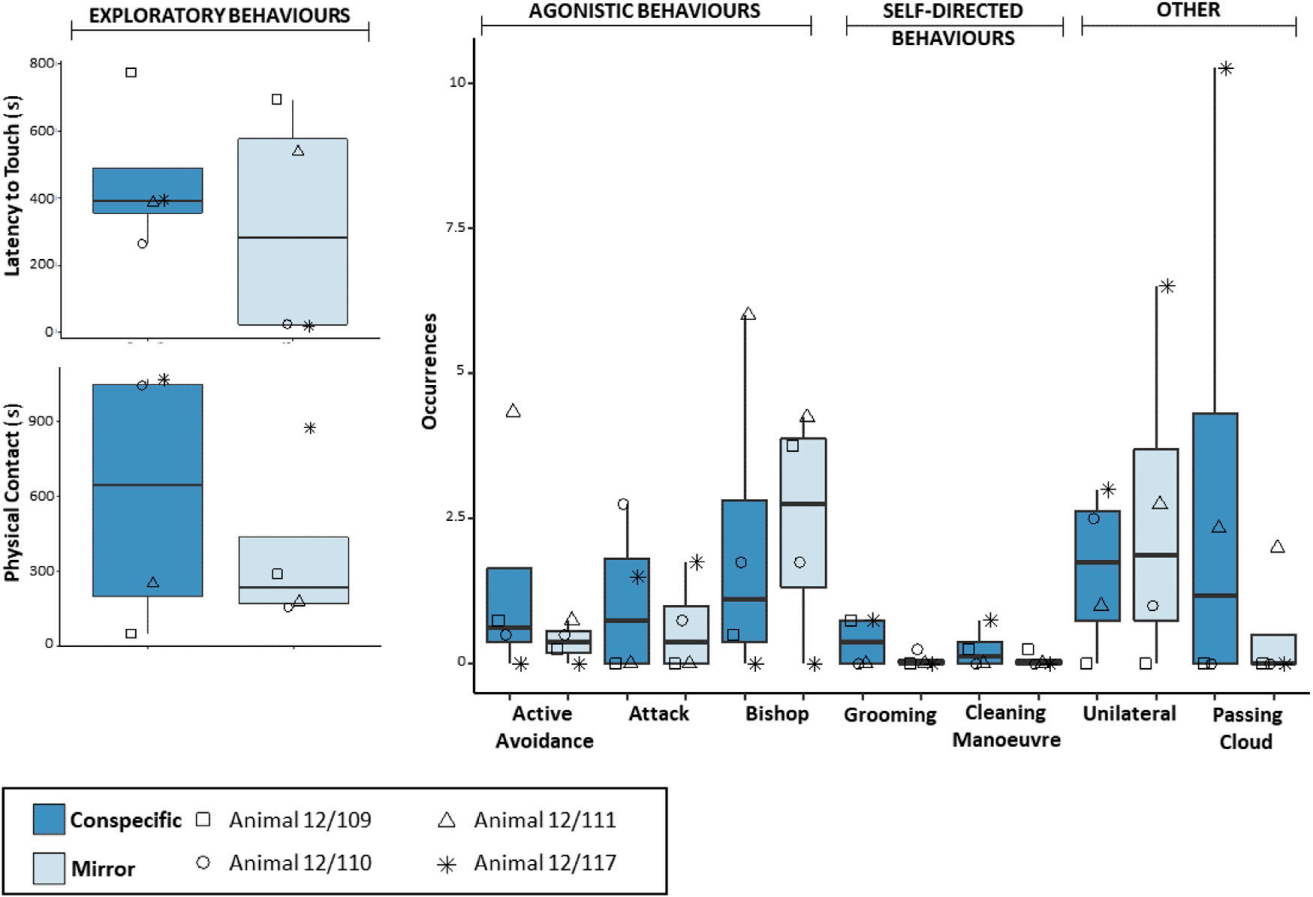
Box and whisker plot of the responses of *O. vulgaris* (*Social* group) to the mirror (light blue) and the conspecific (dark-blue) during the familiarization phase.

Octopuses in the *Social* group exhibited comparable *Latency to touch* in the presence of the mirror and the conspecific, whereas they performed seemingly longer *Physical contact* in trials with the conspecific than in those with the mirror (Figure 3). However, no significant difference between conditions was detected for either exploratory behaviour (Wilcoxon signed-ranked test: *Latency to touch*, *n* = 4, W = −6, *p* = 0.375; *Physical Contact*, *n* = 4, W = −4, *p* = 0.625). Agonistic behaviours were recorded in both conditions, yielding comparable frequencies (Wilcoxon signed-ranked test: *Attack*, *n* = 4, W = −1, *p* = 1; *Active avoidance*, *n* = 4, W = −3, *p* = 0.370; *Bishop*, *n* = 4, W = 1, *p* = 1; Figure 3). Self-directed behaviours were again observed in both conditions, yielding comparable frequencies (Wilcoxon signed-rank test: *Grooming*, *n* = 4, W = −4, *p* = 0.410; *Cleaning manoeuvre*, *n* = 4, W = −1, *p* = 1; Figure 3). The *Unilateral* body pattern was exhibited in the presence of the mirror and the conspecific, yielding comparable frequencies (Wilcoxon signed-rank test: *n* = 4, W = 4, *p* = 0.420; Figure 3). Two individuals performed *Passing cloud* in the social condition and one individual also did so in the presence of the mirror (Supplementary Video S1). No statistical difference was found in the comparison between conditions (Wilcoxon signed-rank test: *Passing cloud*, *n* = 4, W = −3, *p* = 0.370).

### Mark test

Seven out of eight octopuses (including the three sham-marked individuals) groomed their mark and attempted to remove it using their suckers (Table 2). This was typically achieved via a single arm, sometimes following the physical exploration of an area close to the mark, such as the head or the proximal part of one of the first pair of arms (Supplementary Video S2). Notably, in 30 out of 42 instances, mark-directed behaviours were observed in control trials, and thus when the mirror was not present in the tank (Table 2). In only one of the eight paired trials did the octopus (individual 12/109) touch the mark more often in the mirror trial than in the control trial.

**TABLE 2:** Frequency of touches to marks observed in the test.

Individual	Mark	Control Trial	Mirror Trial
12/101	Sham	5	0
12/121	Sham	7	6
12/123	Sham	7	0
12/107	Red	0	0
12/109	Red	0	3
12/110	Red	6	1
12/111	Red	4	2
12/117	Red	1	0

## Discussion

### Familiarization phase

The first aim of this pilot study was to characterize the response triggered by reflected images of self in the common octopus. To this end, we exposed animals to four familiarization trials with a mirror and four familiarization trials with a control stimulus—namely, a non-reflective panel (*Panel* group) or the sight of a conspecific of the same sex and a similar weight (*Social* group). In contrast to the findings in other octopods (Ikeda and Matsumura, 2008; as reported in Ikeda, 2009), our preliminary results indicate that mirror-induced response is variable in the common octopus. We observed frequent physical investigations of the reflective surface and the area behind the surface, as well as attacks, avoidance behaviours, and aggressive displays directed towards the mirror. In the presence of the non-reflective panel, agonistic behaviours were completely absent, whereas exploratory behaviours were overall weaker (e.g., shorter *Physical contact*, less frequent *Behind stimulus*). An exception to the latter result was detected in the performance of one individual. Octopus 12/123 exhibited relatively long *Latency to touch*, minimal *Physical contact*, and never explored the area behind the mirror (*Behind stimulus*). At first blush, these data might be interpreted as a weak response or little interest directed toward the mirror. Yet, when the agonistic response of the animal is taken into account, it seems more likely that the observed limited exploration of the mirror could have been due to a fearful reaction. In fact, high frequencies of *Active avoidance* and *Bishop*, but no *Attack* were scored for octopus 12/123. Given the limited sample size (*n* = 4), it is possible that the performance of this individual—which behaved in differently when compared with others—had a strong influence on the statistical analysis, thus affecting the outcomes and resulting in the non-significant findings in cases where apparent differences between conditions could be detected (i.e., *Physical contact* and *Behind stimulus*; Figure 2).

On the other hand, exploratory and agonistic responses in the *Social* group were comparable between conditions and, in some cases, also consistent at the individual level. For instance, two individuals (e.g., 12/109 and 12/111) never directed attacks toward their reflected image or a conspecific, whereas the remaining two individuals (i.e., 12/110 and 12/117) did so consistently in both conditions. The latter two octopuses were also observed exhibiting shorter *Latency to touch* and longer explorations of one or both stimuli (Figure 3). These data raise the possibility that within each pair, animals established a kind of dominant-subordinate relationship. Such a possibility is consistent with the fact that octopuses 12/110 and 12/117 were never exposed to each other, but rather to individuals 12/111 and 12/109, respectively. Note that, despite being non-gregarious creatures, octopuses have been reported to live at high densities in some sites (e.g., Guerra et al., 2014; Scheel et al., 2016, 2017), form social hierarchies in captivity (Cigliano, 1993), and distinguish between familiar and unfamiliar conspecifics (Tricarico et al., 2011, 2014).

With regard to self-directed behaviours, no clear trend was detected: grooming and cleaning manoeuvres tended to be expressed in low frequencies regardless of whether the animals were being exposed to the mirror, non-reflective panel, or conspecific. It is possible that our data simply represent the normal rates of expression of grooming and cleaning manoeuvres, such that the stimuli used in this study may not have played a key role as triggers. Notably though, the cleaning manoeuvre was displayed in the presence of the mirror only by octopuses 12/123 and 12/109, two individuals that showed an inclination to avoid self-reflected images relative to others. Considering that the cleaning manoeuvre could also function as a displacement activity in common octopus (Packard, 1963), the possibility cannot be excluded that some instances of this behaviour were in fact expressed in response to the stimuli used in the study, particularly if these were perceived as a source of distress. A relevant parallel here may be provided by self-scratching in primates, a displacement activity which is recognized as an indicator of anxiety (Dell’Anna et al., 2022; Troisi, 2002) and which has been observed in response to reflected images of self (Anderson and Gallup, 2015). Ultimately, investigating baseline rates for cleaning manoeuvres in the common octopus could provide insights regarding the interpretation of this behaviour in the presence of a mirror.

Overall, the kinds of mirror-induced responses that we observed in the common octopus match those reported in other coleoids—the physical exploration of the mirror in squid (Ikeda and Matsumoto, 2007) and social displays in decapods (Palmer et al., 2006; Ikeda and Matsumura, 2008; Ikeda, 2009)—and further expand upon them. Thus, our study seems to challenge the idea that the complexity of mirror-induced behaviours correlates with the degree of gregariousness in coleoids, in decreasing order from squids to cuttlefish to octopuses (Ikeda and Matsumura, 2008; Ikeda, 2009). More generally, the repertoire of exploratory, agonistic, and (potentially) self-directed behaviour, as well as inter-individual variability, also resemble those frequently reported among the vertebrates (Plotnik et al., 2006; Anderson and Gallup, 2015; Kohda et al., 2019; Brecht et al., 2020)—at least in the initial phase of exposure to the mirror. However, species that pass the mark test also tend both to perform unusual and repetitive body movements (contingency checking) and use the mirror to inspect otherwise non-visible body parts (self-exploratory behaviour). Throughout the familiarization phase, we could detect no self-exploratory response, though we did notice two unusual and repetitive responses. The first behaviour (here termed “*mantle bobbing*”) resembles a social display described in *Abdopus aculeatus* (i.e., Mantle Bounce Display, Huffard, 2007) and comprised a slow and rhythmic up-and-down movement of the mantle (Supplementary Video S3). The second behaviour (here termed ‘*sweeping’*) comprised a quick, repetitive, and worm-like movement of the distal part of one arm over the reflective surface (Supplementary Video S4). This differed from the more common physical exploration of the stimuli, in which a large part of the ventral surface of the body was kept in contact with mirror. Constant visual contact with the self-reflected image was maintained while the two behaviours were being performed. At this stage, it is not clear whether the *mantle bobbing* and the *sweeping* behaviours constitute true instances of contingency checking. Yet, these behaviours might provide insights about how octopus perceive their reflection, and as such, should be further investigated. To this end, longer familiarization with the mirror may be important, considering that in our experiment the exposure to the stimulus was short (i.e., 120 min) relative to other studies where it lasted several days (e.g., Povinelli et al., 1993; Kohda et al., 2019; Brecht et al., 2020).

### Mark test

The second aim of the present study was to test a procedure for performing the Mark test in the octopus. To this end, sedated animals were marked with a thin layer of soft tissue adhesive overlaid by a layer of non-toxic nail polish. In the test trials, we observed grooming and attempts at removal of the marks both in the absence of the mirror and by sham-marked individuals. Therefore, it is likely that proprioceptive stimuli, rather than visual stimuli, triggered mark-directed behaviour in our experiment. This idea, together with a progressive reduction of mark-directed response, perhaps due to habituation, would also explain the result that a frequency of touches to the mark greater than 70% was observed when the mirror was not present in the tank, namely in the first test trial (Table 2). Alternatively, more complex visual stimuli (e.g., reflected images of self) could have diverted octopus’ attention from any proprioceptive stimulus induced by the marks. Future research should therefore explore alternative marking procedures to test MSR in cephalopods. The use of elastomers as marks might provide a valuable option, given that these subcutaneous tags have been successfully employed to monitor octopus populations in the wild (Brewer and Norcross, 2012), as well as to conduct the Mark test in fish (Kohda et al., 2019).

In conclusion, the present study shows that common octopuses display a distinct and varied repertoire of mirror-induced responses and, we believe, provides preliminary baseline data for further exploration of MSR in this species. Despite the shortcomings of the marking procedure we used here, results obtained in our test trials demonstrate that octopuses are capable of performing mark-directed responses and that the Mark test offers a suitable paradigm for investigating MSR in these animals. However, because many of the responses we observed were not visually mediated, it would be important for future research to include additional controls in the Mark test, including measurement of the frequency of physical exploration of an unmarked area of the body. In addition, another critical next step would be to provide longer exposure to the mirror during the familiarization phase. This would both provide an opportunity to explore variability of mirror-induced behaviour over time and afford further insights into how octopus perceive their own reflections.

## Data Availability

The original contributions presented in the study are included in the article/Supplementary Material, further inquiries can be directed to the corresponding author.
